# Brain AT1 and AT2 receptors and nitric oxide in baroreflex regulation of renal sympathetic activity in unanaesthetised rats

**DOI:** 10.1038/s41598-025-26231-6

**Published:** 2025-11-26

**Authors:** Mohammed H. Abdulla, Edward J. Johns

**Affiliations:** Department of Physiology, Western Gateway Building, University College Cork, College Road, Cork, Ireland

**Keywords:** Baroreflex, Renal sympathetic nerve activity, PD123319, Losartan, Angiotensin II, AT2, Maximum gain, Cardiology, Neurology, Neuroscience, Physiology

## Abstract

This study investigated the role of brain AT1 and AT2 receptors and the nitric oxide (NO) system in modulating renal sympathetic nerve activity (RSNA) baroreflex in unanaesthetised rats. Baroreflex gain curves (BRC) were generated following intracerebroventricular (I.C.V.) infusion of saline, Ang II, or Ang II combined with either losartan, PD123319 (AT2 antagonist), or L-NAME (NO synthase inhibitor). An AT2 agonist (CGP42112) was also infused I.C.V. with L-NAME. RSNA baroreflex sensitivity increased by 60% (*P* = 0.004) following losartan compared to saline (-4.8 ± 1.9 vs. -3.0 ± 0.9), but not after CGP42112. I.C.V. Ang II increased maximum gain by ~ 70% (*P* = 0.003) compared to saline (-5.0 ± 1.6 vs. -3.0 ± 0.9). This effect was reversed when Ang II was co-infused with PD123319 (-3.7 ± 1.1), but not losartan. I.C.V. CGP42112 increased the overall response range of the baroreflex but lowered the minimum level it could reach compared to saline (*P* = 0.03 − 0.02). The baroreflex effects of I.C.V. CGP42112 (*P* = 0.013), but not Ang II, were abolished when co-infused with L-NAME. These findings demonstrate an important facilitatory role for AT2 in baroreflex regulation of RSNA in unanaesthetised rats at basal brain levels of Ang II, a mechanism that is dependent on a functional NO system. By contrast, AT1 exerts an inhibitory effect on the baroreflex that is independent of NO. These observations suggest that targeting central AT2 receptors may represent a potential therapeutic strategy for conditions such as neurogenic hypertension, where impaired baroreflex function is present.

## Introduction

The brain renin-angiotensin system (RAS) plays a critical role in cardiovascular regulation by modulating sympathetic nerve activity, influencing blood pressure, and contributing to homeostatic control of the circulation. This system comprises various neuropeptides, particularly angiotensin II (Ang II), which influences blood pressure and vascular tone. Ang II, through the activation of angiotensin II type 1 (AT1) receptors, promotes increased blood pressure by enhancing sympathetic outflow^1,2^ and hence vascular resistance. Conversely, the brain angiotensin II type 2 (AT2) receptor exerts a counter-regulatory effect, attenuating the hypertensive influence of AT1 receptor activation^3,4^. Notably, studies have demonstrated that pharmacological blockade of AT1 receptors in rat brain vessels^5^ enhances the compensatory AT2-mediated vasodilatation. Similarly, AT2 gene deletion in mice enhances the pressor effect of Ang II via AT1 receptors^6^. Furthermore, selective central AT2 receptor activation has been shown to lower blood pressure and improve baroreflex sensitivity in heart failure^7^ and spontaneously hypertensive^8^ rats. These findings highlight the interplay between brain AT1 and AT2 receptors in the regulation of sympathetic activity and arterial blood pressure.

The mechanism of the centrally mediated cardiovascular changes due to activation of AT2 receptors is not fully understood, particularly in terms of its interactions with AT1 receptors and the extent to which nitric oxide (NO) signalling contributes to its cardiovascular effects. However, accumulating evidence suggests that AT2 receptor-mediated blood pressure regulation is closely linked to NO signalling^9,10^. Previous studies have demonstrated that increased nitric oxide synthase (NOS) activity is associated with decreased neuronal AT1 receptor expression^11^, whereas NO inhibits the effects of brain AT1 receptor activation on renal sympathetic nerve activity, mean arterial pressure, and heart rate^12^. Similarly, the cardiovascular and baroreflex-modulating effects of brain AT2 receptors appear to be NO-dependent^8,13^. These findings indicate a complex interplay between AT1/AT2 receptor regulation and NO signalling. Nonetheless, the role of Ang II AT1/AT2 receptor activation in the brain, particularly in the modulation of baroreflex control of blood pressure in unanaesthetised rats, remains inadequately characterized, and it is unclear whether these effects are mediated by NO signalling. Studying unanaesthetised rats is crucial because it allows for the assessment of baroreflex function under physiological conditions, avoiding the confounding effects of anaesthesia on autonomic and cardiovascular responses.

In this study, mechanisms were explored by which AT1/AT2 receptor activation in the brain influences autonomic function and blood pressure control. Specifically, the investigation evaluated the effects of exogenous Ang II administration I.C.V. on baroreflex sensitivity and assessed the potential role of NO in mediating these responses. The hypothesis tested was that AT2 receptor stimulation enhances baroreflex sensitivity through an NO-dependent mechanism, as previous studies have shown that central AT2 receptor activation promotes NO production, which in turn facilitates vasodilation and autonomic regulation.

## Results

### Steady-state values of MAP, HR and integrated RSNA

Steady-state levels of MAP, HR, and RSNA measured immediately before baroreflex gain curve generation in all groups are shown in Table 1. The I.C.V. administration of Ang II significantly increased MAP (*p* = 0.002) and decreased RSNA (*p* = 0.021) compared to I.C.V. saline, without significantly affecting HR. Meanwhile, I.C.V. injection of Ang II in the presence of the AT2 receptor blocker PD123319 (Ang II/Ang II + PD group) was associated with a significantly increased MAP (*p* = 0.006) but with no changes in either HR or RSNA.

The I.C.V. injection of the AT2 receptor agonist CGP42112 (Saline/CGP group) produced a significant increase in HR (*p* = 0.031), without changes in MAP or RSNA. This pattern was mirrored in the Ang II/Ang II + LOS group, where HR was significantly increased (*p* = 0.004) when Ang II effects on AT2 receptors were unmasked by blocking AT1 receptors. In the Saline/LOS group, losartan infusion did not significantly alter MAP, HR, or RSNA.

NOS inhibition with L-NAME (LNM) significantly increased both MAP (*p* = 0.001) and HR (*p* = 0.002) without significantly altering RSNA in the Saline/LNM group. In the Ang II/Ang II + LNM group, NOS inhibition further augmented MAP (*p* = 0.004), with no significant changes in HR or RSNA. Similarly, in the CGP/CGP + LNM group, MAP and HR significantly increased (*p* = 0.008 and *p* = 0.029, respectively), with no changes in RSNA.

### Baroreflex curves of RSNA

**Fig. 1:**
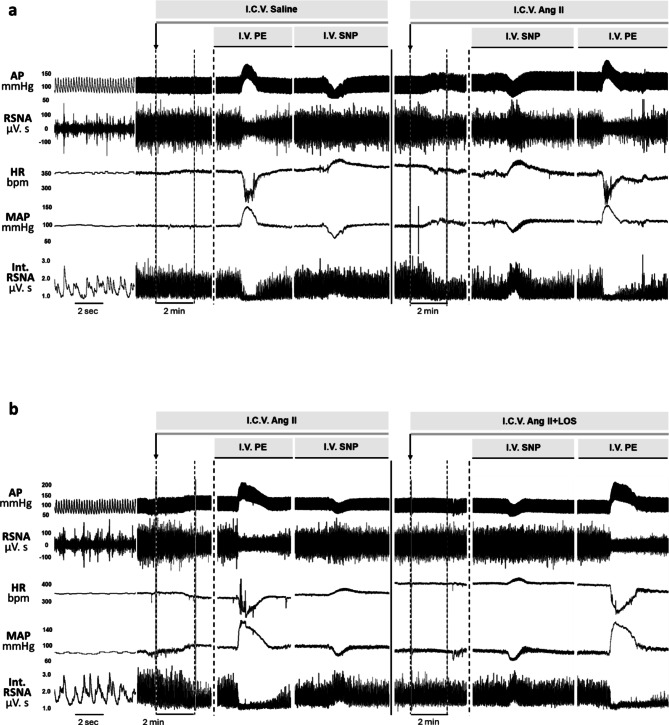
Sample data from a baroreflex gain experiment. In the presence of a background I.C.V. infusion of either saline, Ang II or Ang II + Losartan, the baroreflex was tested by measuring reflex changes in RSNA in response to MAP changes induced by I.V. infusions of Phenylephrine (PE) and sodium nitroprusside (SNP). Representative examples show original recordings of pulsatile arterial pressure (AP), MAP, HR, and raw and integrated RSNA in a rat infused with Ang II (a) and a rat infused with Ang II + Losartan (b) I.C.V. HR, heart rate; MAP, mean arterial pressure; RSNA, renal sympathetic nerve activity; Ang II, angiotensin II; LOS, losartan.

Figure 1 illustrates the raw and integrated recordings of RSNA and HR at baseline and during ramp changes in arterial pressure induced by intravenous PE and SNP infusions in one rat infused with I.C.V. Ang II (Fig. 1a), and in another rat infused with Ang II plus losartan I.C.V. (Fig. 1b). PE infusion caused an increase in MAP of approximately 50 mmHg, accompanied by a corresponding decrease in RSNA and HR. Conversely, SNP infusion led to a marked decrease in MAP by about 50 mmHg, which triggered a parallel increase in RSNA and HR.

**Fig. 2:**
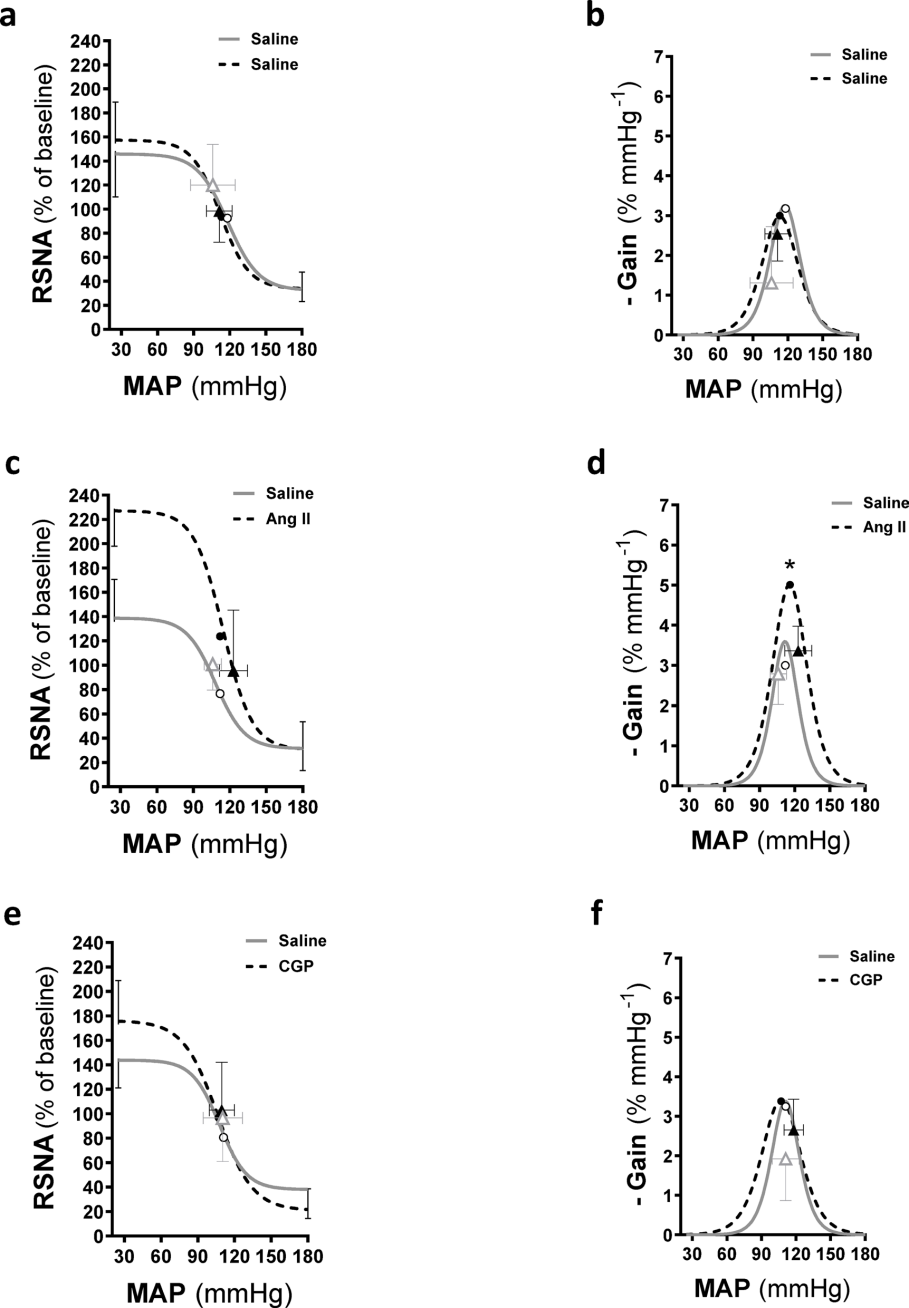
Average baroreflex gain curves and maximal gain of renal sympathetic nerve activity (RSNA) following ramp changes in mean arterial blood pressure in I.C.V. saline infused rats followed by saline (a, b), Ang II (c, d) and CGP (e, f). *P < 0.05, second baroreflex maximum gain compared with first baroreflex maximum gain. Symbols on the curves represent steady-state MAP values measured immediately before the baroreflex (open and closed triangles) and mid-point MAP (open and closed circles). RSNA, renal sympathetic nerve activity; MAP, mean arterial pressure; Ang II, angiotensin II; CGP, CGP42112.

**Fig. 3:**
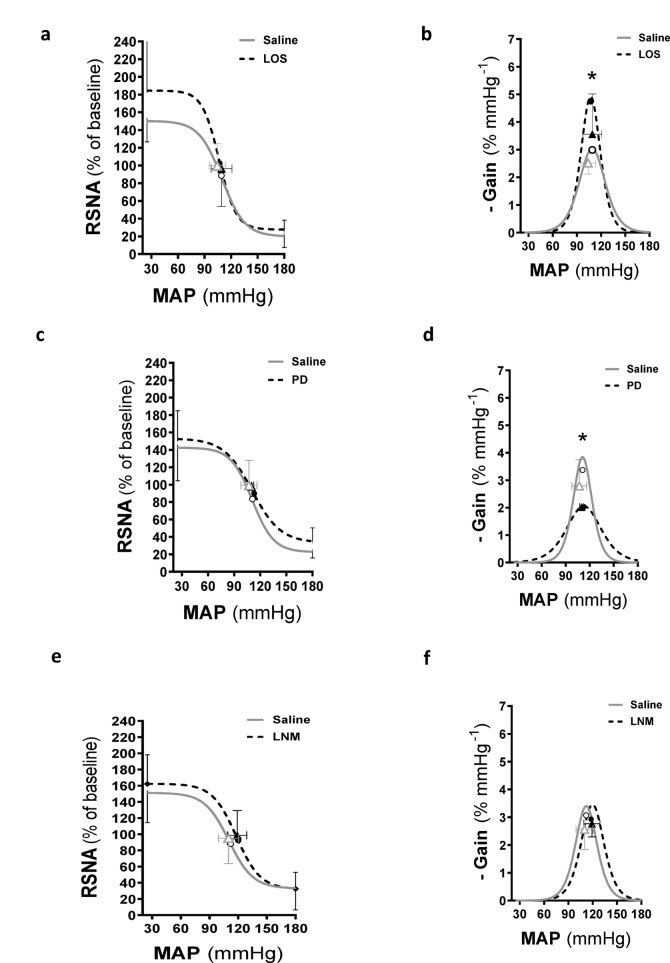
Average baroreflex gain curves and maximal gain of renal sympathetic nerve activity (RSNA) following ramp changes in mean arterial blood pressure in I.C.V. saline infused rats followed by losartan (a, b), PD (c, d) and L-NAME (e, f). *P < 0.05, second baroreflex maximum gain compared with first baroreflex maximum gain. Symbols on the curves represent steady-state MAP values measured immediately before the baroreflex (open and closed triangles) and mid-point MAP (open and closed circles). RSNA, renal sympathetic nerve activity; MAP, mean arterial pressure. LNM, L-NAME; LOS, losartan; PD, PD123319.

Tables 2, 3 and 4; Figs. 2 and 3 present the parameters and average constructed baroreflex gain curves for the relationship between RSNA and MAP following I.C.V. administration of saline during the control phase, and subsequently after I.C.V. administration of Ang II, CGP, losartan, PD, and L-NAME. In the time control group (saline/saline), all baroreflex gain curve parameters (Table 2; Fig. 2a, b) following I.C.V. saline in the first phase were comparable to those observed after I.C.V. saline administration in the second phase. The I.C.V. Ang II infusion was associated with expanded range (*A*1), as well as increased sensitivity (maximum response and maximum gain) (all *p* ≤ 0.003; Table 2; Fig. 2c, d). By contrast, I.C.V. CGP did not significantly alter baroreflex gain curve parameters compared to saline, except for expanded range (*A*1, *p* = 0.029) and a drop in the lowest point of the curve (*A*4, *p* = 0.017) (Table 2; Fig. 2e, f).

The baroreflex gain curve parameters in the presence of I.C.V. losartan showed increased sensitivity (*A*2, *p* = 0.037) and greater maximum gain (*p* = 0.004) compared to saline. However, the MAP saturation and operating range were both lower than those observed with saline (both *p* < 0.05; Table 3; Fig. 3a, b). The I.C.V. infusion of PD was associated with a lower sensitivity (*A*2), MAP threshold and maximum gain (all *p* = 0.002–0.006) but higher MAP saturation (*p* = 0.007) and operating range (*p* = 0.002) compared with saline (Table 3; Fig. 3c, d). The baroreflex gain curve parameters in the presence of I.C.V. L-NAME demonstrated a higher mid-point pressure (A3) (*p* = 0.025) but no changes in any of the other baroreflex gain curve parameters (Table 3; Fig. 3e, f).

**Fig. 4:**
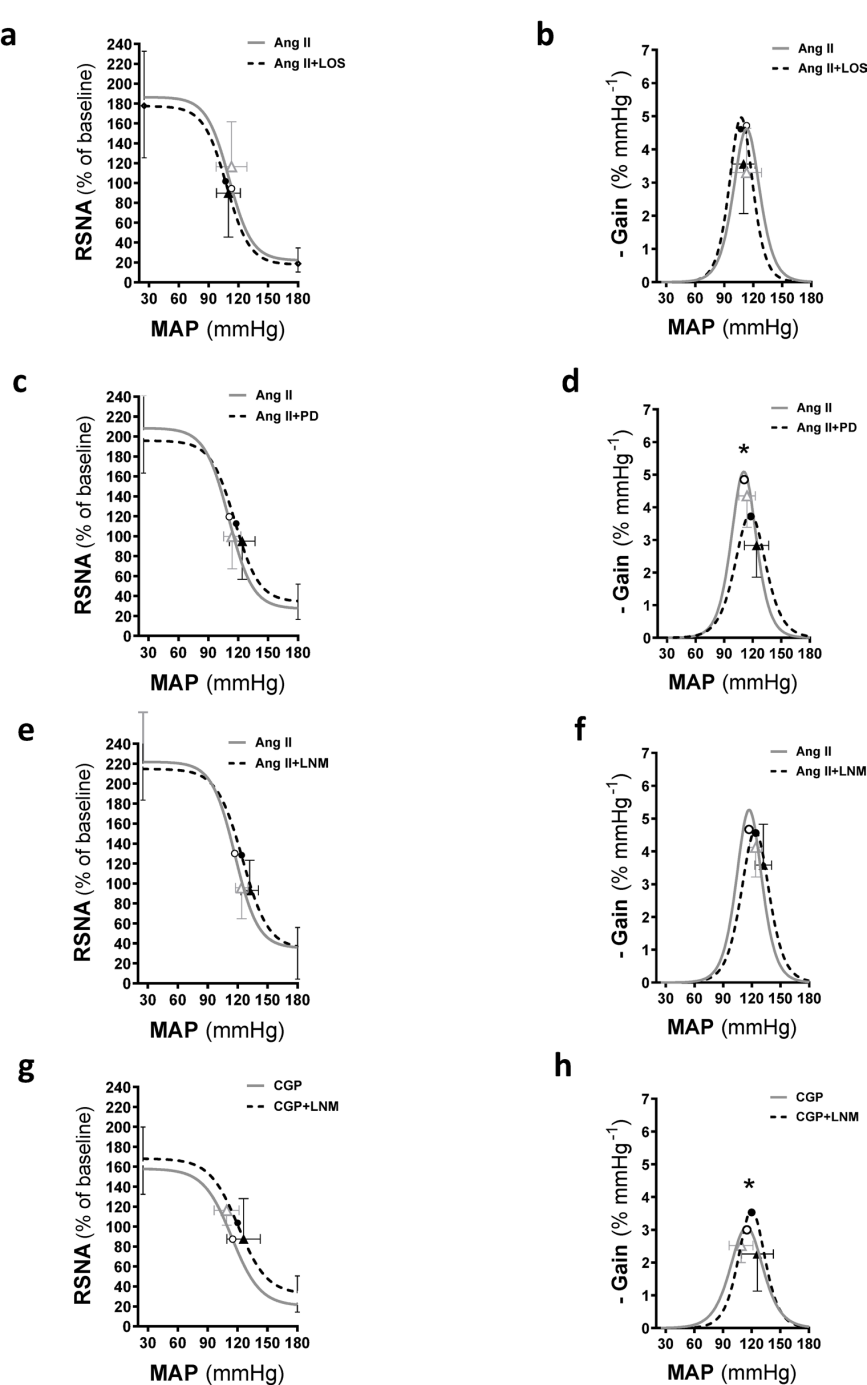
Average baroreflex gain curves and maximal gain of renal sympathetic nerve activity (RSNA) following ramp changes in mean arterial blood pressure in I.C.V. Ang II infused rats followed by Ang II + losartan (a, b), Ang II + PD (c, d) and Ang II + LNM (e, f). CGP was infused I.C.V. followed by CGP + LNM (g, h). *P < 0.05, second baroreflex maximum gain compared with first baroreflex maximum gain. Symbols on the curves represent steady-state MAP values measured immediately before the baroreflex (open and closed triangles) and mid-point MAP (open and closed circles). RSNA, renal sympathetic nerve activity; MAP, mean arterial pressure. LNM, L-NAME; LOS, losartan;  Ang II, angiotensin II; CGP, CGP42112; PD, PD123319.

Tabl﻿e 4; Fig. 4 show the parameters and average baroreflex gain curves representing the relationship between RSNA and MAP following I.C.V. administration of Ang II during the control phase, and following I.C.V. administration of Ang II combined with either losartan, PD, or L-NAME. All baroreflex gain curve parameters following I.C.V. Ang II plus losartan in the second phase were comparable to those observed after I.C.V. Ang II alone in the first phase (Table 4; Fig. 4c, d). However, I.C.V. administration of Ang II in combination with PD resulted in a lower sensitivity (*A*2) and maximum gain (*p* = 0.03), but a higher mid-point pressure (*A*3), MAP saturation, and operating range (*p* = 0.009–0.05) (Table 4; Fig. 4c, d). The baroreflex gain curve parameters during I.C.V. injection of Ang II plus L-NAME were similar to those recorded with Ang II alone (Table 4; Fig. 4e, f). Furthermore, I.C.V. administration of CGP combined with L-NAME was associated with increased sensitivity (*A*2), greater maximum gain, and an elevation in the lowest point (*A*4) of the baroreflex curve (*p* = 0.01–0.05) (Table 4; Fig. 4g, h).

## Discussion

The present study demonstrates that central AT2 receptors play a key facilitatory role in modulating the RSNA baroreflex response to acute changes in arterial pressure in unanaesthetised rats. Under physiological conditions, when AT1 receptors are blocked, AT2 receptor activation by brain Ang II enhances baroreflex sensitivity—evident in increased slope (*A*2) and maximum gain of the RSNA–MAP relationship. This indicates a context-dependent modulatory role for AT2 receptors, in contrast to the inhibitory influence exerted by AT1 receptor activation by both brain and I.C.V. infused Ang II. Importantly, when AT2 receptor activity was pharmacologically inhibited with PD123319, the baroreflex-enhancing effects of I.C.V. infused Ang II—particularly increased gain and range—were blunted. Conversely, selective AT2 receptor activation with CGP42112 enhanced the baroreflex operating range and lowered the minimum point of the baroreflex gain curve, effects that were abolished by NOS inhibition. These data provide further support for the view that AT2-mediated facilitation of baroreflex function is NO-dependent, whereas the pressor and baroreflex effects of I.C.V. injections of Ang II itself appear independent of NOS. This dissociation underscores distinct downstream pathways for AT1 and AT2 receptor signalling in central cardiovascular control.

Our findings also highlight a divergence in the central mechanisms governing arterial versus cardiopulmonary baroreflexes. Unlike our previous report, in which volume expansion responses were modulated only after exogenous AT2 activation by CGP42112^14^, the current study demonstrates that the high-pressure baroreflex is enhanced following AT2 stimulation by brain Ang II. This suggests that distinct neural circuits or receptor distributions mediate these two reflex types.

The primary aim of this study was to elucidate the mechanisms through which central AT1 and AT2 receptor activation contributes to the arterial pressure baroreflex responses, particularly in the absence of confounding effects from anaesthesia. Direct I.C.V. administration of pharmacologic agents was validated by physiological responses such as thirst and pressor responses. Furthermore, baroreflex sensitivity was quantified using both range-dependent (maximum gain) and range-independent (slope) parameters.

Previous studies have indicated an important role of brain AT1 and AT2 receptors in the modulation of sympathetic outflow and blood pressure regulatory mechanisms^8,15,16^. For instance, AT1 receptors in the medial amygdaloid nucleus neurons were shown to facilitate baroreflex responses during stress^16^. Similarly, chronic stimulation of central AT2 receptors enhanced the spontaneous baroreflex sensitivity and reduced blood pressure in hypertensive rats^8^. Furthermore, Ang II pressor effects were enhanced in AT2 receptor knockout mice, although they had normal resting blood pressure^17^. On the other hand, Ang II vascular effects were abolished in transgenic mice with overexpression of AT2 receptors^18^. Together, these studies point to a potential blood pressure control mechanism mediated by activation of Ang II receptors.

This is one of the first demonstrations that activation of central AT2 receptors by brain Ang II augments high-pressure arterial baroreflex function in unanaesthetised rats. However, the activation of AT2 by I.C.V. infusion of CGP42112 contributes only minimally to the RSNA baroreflex response to changes in arterial pressure. These findings are supported by previous studies in unanaesthetised rats demonstrating minimal changes in the baroreflex following activation of brain AT2 receptors using I.C.V. infusion of agonists such as CGP42112A^19^ and Compound 21^7^.

Direct I.C.V. administration of Ang II significantly increased MAP and decreased RSNA, without affecting HR, consistent with activation of central AT1 receptors that mediate a pressor response and sympathoinhibition^20–22^. In contrast, central infusion of losartan alone did not significantly alter MAP, HR, or RSNA, corroborating previous findings in rats^23^. This suggests that AT1 receptors do not exert tonic effects under baseline physiological conditions. However, co-administration of Ang II with losartan centrally significantly increased HR suggesting that AT1 receptor blockade unmasks a chronotropic effect likely mediated by AT2 receptor activation. This interpretation is supported by Zhu et al.^24^, who showed that the HR increase induced by Ang II plus losartan was reversed by AT2 receptor blockade with PD123319. Additionally, selective activation of central AT2 receptors with CGP42112 in the present study resulted in a similar HR increase without affecting MAP or RSNA.

To further delineate the role of AT2 receptors, we examined the effects of Ang II infusion during AT2 blockade. Co-administration of Ang II and PD123319 was associated with a significant increase in MAP, with no changes in HR or RSNA. This supports the hypothesis that AT2 receptors normally exert a buffering, counterregulatory effect against AT1-mediated increases in MAP^25^. This interpretation is consistent with findings by Siragy et al.^26^, who reported exaggerated hypertensive responses to Ang II in AT2 receptor–deficient mice compared to wild-type controls.

Inhibition of central NOS with L-NAME elevated both MAP and HR without significantly altering RSNA, consistent with a tonic vasodilatory role for central nitric oxide^27^. When Ang II was administered during NOS inhibition, MAP was further elevated, again without significant changes in HR or RSNA. These data suggest that nitric oxide acts centrally to restrain the pressor effects of Ang II^28,29^. Moreover, selective activation of AT2 receptors in the presence of L-NAME also led to increased MAP and HR, indicating that the cardiovascular effects of central AT2 receptor stimulation are NO–dependent^30^.

This study provides new evidence for the modulatory role of central Ang II receptors and NO in regulating the arterial baroreflex in unanaesthetised rats. At basal brain levels of Ang II, central AT2 receptor activation enhanced baroreflex sensitivity, increasing both the slope (*A*2) and maximum gain of the response. These findings align with previous observations in conscious rats^19^. In contrast, AT2 receptor blockade with PD123319 revealed that activation of AT1 receptors by brain Ang II exerts an inhibitory effect on baroreflex function. This was evidenced by a reduced baroreflex gain and slope (*A*2), along with an expanded operating range and elevated lower plateau. These findings reinforce the concept that under physiological conditions, AT1 receptors dampen baroreflex sensitivity, consistent with our earlier results in anaesthetised rats^13^.

Central NOS inhibition with L-NAME in the present study increased the baroreflex mid-point pressure without affecting overall baroreflex gain. These findings are consistent with a previous study in conscious sheep, where the maximum gain of the RSNA baroreflex remained unchanged following I.C.V. injection of L-NAME^31^. Conversely, Matsumura et al.^32^, showed an enhanced baroreflex sensitivity to I.C.V. L-NAME in conscious rabbits. These conflicting results point to a possible species-specific effects of central NOS system and to the position of the I.C.V. cannula.

When Ang II was infused I.C.V., baroreflex sensitivity increased, primarily due to an expanded response range rather than changes in gain slope. This pattern matches prior studies in conscious rabbits^33,34^. Notably, the baroreflex enhancement induced by I.C.V. infusion of Ang II persisted despite AT1 receptor blockade with losartan but was reversed by PD123319. These results indicate that the baroreflex effects of exogenously administered Ang II observed in this study are partly mediated through central AT2 receptors. This is further supported by the finding that CGP42112, a selective AT2 receptor agonist, increased range and reduced the lower plateau of the baroreflex gain curve . The dependency of AT2 receptor–mediated baroreflex enhancement on nitric oxide was confirmed by the loss of CGP42112 effects on the baroreflex lower plateau following NOS inhibition. Our results are consistent with a previous study using Compound 21 to centrally activate AT2 receptors, which led to reductions in blood pressure and norepinephrine excretion via nNOS/NO signalling within the paraventricular nucleus (PVN) and rostral ventrolateral medulla (RVLM)^35^.

The present study examined the overall central contribution of AT1 and AT2 receptors and nitric oxide to baroreflex regulation. However, site-specific approaches remain essential for delineating the distinct roles of individual baroreflex-related brain regions such as the PVN, RVLM, and nuclear tractus solitarius (NTS). Consistent with our findings, microinjection studies in anaesthetised rats have shown that injection of Ang II either into the PVN or NTS is associated with increased MAP and HR^36–38^. Moreover, although microinjection of PD123319 into the PVN had no effect on MAP or HR, it attenuated both blood pressure and single-unit responses to Ang II, supporting a role for Ang II acting via AT2 receptors^39^. The RVLM has also been shown to play a major role in baroreflex responses to I.C.V. Ang II. Studies by the Head et al. group^38^ in conscious rabbits demonstrated baroreflex gain curve changes following RVLM injection of Ang II that are comparable to those observed in the present study. Thus, while our global I.C.V. approach provides an integrated view of the central contribution of different brain regions to baroreflex regulation, site-specific investigations in conscious animals remain essential for defining the magnitude and extent of the individual contributions of these regions.

Reactive oxygen species (ROS) influence multiple signalling pathways, including the renin–angiotensin system (RAS), thereby affecting cardiovascular and renal function. In the brain, RAS has been shown to modulate blood pressure through interactions with ROS in the PVN, contributing to the development of hypertension. The mechanism underlying RAS–ROS interactions in the PVN has been proposed to involve the AT1 receptor/PKCγ/Rac1 pathway, which induces ROS overproduction via an NAD(P)H-dependent mechanism, ultimately leading to sympathoexcitation and increased blood pressure^40^. Similarly, ROS have been implicated in elevated sympathetic nerve activity by diminishing NO-mediated effects in the RVLM^41^. These findings suggest that part of the effects of I.C.V.-administered Ang II may be mediated through ROS in brain regions directly involved in the baroreflex regulation of blood pressure^42^. Conversely, AT2 receptors are closely associated with nNOS in the NTS, suggesting that their antagonistic influence on AT1 receptor activity may be mediated through nNOS-derived NO production, which counteracts ROS^43^. This interpretation is further supported by our current and previous findings showing that the CGP42112-mediated enhancement of baroreflex function was abolished by L-NAME. While the present study did not directly assess ROS, consideration of these mechanisms provides additional context for interpreting the differential contributions of AT1 and AT2 receptors to baroreflex control.

In summary, our findings reveal a mechanism by which central AT2 receptor activation enhances arterial baroreflex function under physiological levels of Ang II, an effect that is NOS–dependent. In contrast, AT1 receptor activation when AT2 receptors are blocked suppresses baroreflex sensitivity. Notably, I.C.V. infusion of Ang II enhances baroreflex responses through AT2 receptors. These results underscore the distinct and opposing roles of AT1 and AT2 receptors and the context-dependent nature of Ang II actions in central cardiovascular regulation.

## Limitations

First, although we employed unanaesthetised rats to avoid the confounding effects of anaesthesia on baroreflex function, this experimental paradigm introduces the possibility of elevated basal sympathetic drive due to surgical stress and placement in a plexiglass holder. However, all RSNA and cardiovascular parameters were comparable to those reported in our previously published studies using anaesthetised rats^44–46^. Moreover, this experimental approach has been widely adopted in prior studies, demonstrating minimal impact on basal renal and cardiovascular function parameters^47–50^.

Second, the use of I.C.V. drug administration does not allow for precise localization of receptor-mediated effects within specific brain regions. Therefore, the exact neural circuits and nuclei involved remain to be identified. Wainford and Kapusta^51^ using the same coordinates as in this study, suggested that the PVN and RVLM are likely targeted by this I.C.V. approach. Third, although pharmacological agents such as losartan, PD123319, CGP42112, and L-NAME were chosen for their receptor specificity, off-target effects or incomplete selectivity cannot be entirely excluded, particularly at higher doses. In addition, while systemic spillover of centrally administered agents was minimized, it may still influence peripheral responses and confound interpretation of central mechanisms. Notably, a small injection volume and short infusion period were used in this study to mitigate such off-target or peripheral effects. Fourth, although the study design distinguishes between the effects of brain vs. I.C.V. Ang II, the concentrations achieved through I.C.V. infusion may not fully replicate physiological or pathophysiological conditions, thereby limiting the translational relevance of these findings to human diseases such as hypertension or heart failure. Furthermore, we did not assess the potential contribution of Ang III. Previous studies have shown that Ang II is rapidly converted to Ang III in the brain, and that Ang III may act as the primary effector at AT2 receptors^52–54^. Thus, it remains possible that some of the responses observed following I.C.V. Ang II administration were mediated, at least in part, by Ang III. Future studies directly measuring central Ang III levels will be required to clarify its role in the effects described here. Finally, this study was conducted exclusively in male rats. Given known sex differences in baroreflex regulation^55^ and angiotensin receptor expression^56^, the generalizability of these findings to females remains uncertain and warrants further investigation.

## Methods

### Animals

All experimental procedures adhered to the European Community Directive 86/609/EC and received approval from the Animal Examination Ethical Committee at University College Cork (Approval number: 2012-025). Male Wistar rats (300–350 g body weight) were obtained from Harlan (Bicester, UK) and housed in the Biological Services Unit, Cork, Republic of Ireland. Animals were provided tap water and standard irradiated rodent normal salt (0.3%) diet *ad libitum* (Harlan-Teklad, Bicester, UK). To ensure acclimatization, they were housed for at least one week before the commencement of experimental procedures. During this period, the rats were kept under a controlled 12-hour light-dark cycle at an ambient temperature of 20 ± 3 °C with 40–70% humidity. We confirm that the study is reported in accordance with ARRIVE guidelines (https://arriveguidelines.org).

### Chronic implantation of intracerebroventricular (I.C.V.) cannula

Five to seven days before experimentation, rats were anaesthetized with an intraperitoneal injection of a combination of ketamine (30 mg/kg) and xylazine (3 mg/kg) anaesthetics^57,58^. Additionally, rats were injected with SC carprofen (5 mg/kg), a non-steroidal anti-inflammatory drug, to provide analgesia during and after the experiment. A stereotaxic frame was used to implant a stainless steel cannula into the right lateral cerebral ventricle using the following co-ordinates: 0.3 mm posterior to the bregma, 1.3 mm lateral to the midline and 4.5 mm below the skull surface as previously described^59–61^. The cannula was fixed in place with jeweller’s screws (~ 1 mm in diameter and ~ 2.5 mm in length) and dental cement. Animals were kept warm using heated pads placed under their home cages until they recovered from the anaesthesia and were allowed to recover for 4–5 days before the acute experiment.

### Acute experiment surgical procedure

On the day of the experiment, rats were anaesthetized with intraperitoneal tribromoethanol (250 mg/kg; Sigma-Aldrich, UK). Polyethylene catheters (PE-25 tubing connected to PE-50; Smiths Medical International Ltd., Kent, UK) were implanted into the left femoral artery and vein for continuous mean arterial pressure (MAP) and heart rate (HR) recording, and isotonic saline infusion (150 mM NaCl; 2 ml/h), respectively.

In order to record renal sympathetic nerve activity in these rats, the left kidney was exposed via a retroperitoneal flank incision. A renal nerve bundle was then isolated and carefully dissected free under a dissecting microscope (Leica Wild M420, Ashbourne, Ireland). The nerve bundle was placed on a bipolar stainless steel wire electrode attached to a high-impedance head stage, which was coupled to an amplifier (NeuroAMP EX, ADInstruments, UK) and connected to a PowerLab data acquisition system (ADInstruments, UK). The recorded renal sympathetic nerve signals were relayed to both an audio amplifier and the data acquisition system. Once a clear signal was identified, the nerve bundle and recording electrode were secured in place using dental impression material (President, Coltène, Altstätten, Switzerland). The electrode cable was exteriorized at the back of the animal and the incisions were closed and sutured using surgical thread. Finally, rats received SC carprofen (5 mg/kg) to provide analgesia.

After surgical preparation, the rats were placed in a custom-made Plexiglass rat holder to minimize movement and prevent loss of renal nerve activity due to motion^47,62^. The rat holder allowed the rat forward and backward movement and did not restrain the rat. Additionally, animals were provided access to tap water during the experiment, along with a maintenance IV saline infusion (2 ml/h). The I.C.V. cannula was connected via a polyethylene tube (PE-10, Portex, UK) to a 25-µL Hamilton microsyringe (Hamilton, USA) fitted to a microinfusion pump (KD Scientific, Linton Instruments, UK). The arterial cannula was attached to a pressure transducer, which was connected to an amplifier (ADInstruments, UK). The experimental protocol was initiated 2.5 h after surgery, once the animals had fully recovered from anaesthesia, ensuring stable RSNA recording and cardiovascular function prior to experimentation.

### Direct measurement of renal sympathetic nerve activity (RSNA)

A multifibre direct RSNA recording was performed in these unanaesthetised rats using recording electrodes connected to a low-noise, high-gain signal amplifier (NeuroAmp EX^®^, ADInstruments, Hastings, UK). RSNA was amplified (×10,000) and filtered (high-pass: 100 Hz; low-pass: 2 kHz). The integrated RSNA signal was analysed using LabChart 7.0 software (ADInstruments, UK) to assess the renal sympathetic nerve activity baroreflex gain curve.

### Acute experiment protocol

Studies were performed once the animals had recovered from anaesthesia and MAP, HR, and RSNA had stabilised. The complete experimental protocol for I.C.V. infusions and baroreflex testing lasted an average of 2.5 h. The I.C.V. cannula was attached to the micropump, and infusions of either vehicle (saline) or drugs were initiated for 2 min at 1 µl min^− 1^, followed by a maintenance infusion of 5 µl h^−1  ^ for 15 min. During this period, the first baroreflex gain curve was generated by I.V. infusion of phenylephrine (PE) and sodium nitroprusside (SNP) to increase and decrease blood pressure, respectively. Each drug was administered at a dose of 10 µg in 0.2 mL of saline, infused at a rate of 18 mL h^− 1^ over 40 s.

A time period of 5–10 min was allowed following the infusion of phenylephrine and sodium nitroprusside for all the variables to return to baseline. After the first baroreflex challenge, a recovery period of at least 30 min was provided before the second I.C.V. drug administration, after which baroreflex curve generation for RSNA was repeated in a similar manner. The I.C.V. maintenance infusion of saline or drug was continued throughout the baroreflex curve generation period, and haemodynamic and RSNA data were continuously collected throughout the study. At the end of the acute experimental protocol, the animals were euthanised using an intravenous overdose of anaesthetic^63,64^. Death of animals was confirmed by lack of a heartbeat and lack of respiration. The background noise value, measured 30 min after euthanasia injection, was subtracted from all original recordings of the integrated signal and used for baroreflex gain curve analysis.

## Experimental groups

Ten groups of rats were used in this study:

1. *Saline-controlled groups*: The first five groups received saline as the first I.C.V. infusion at a rate of 1 µl min^−1^ for 2 min as a loading dose, followed by a maintenance dose of 5 µl h^−1^. The second I.C.V. infusion was then switched to either saline (*n* = 6); CGP42112 (CGP, 50 µg kg^−1^ min^−1^, *n* = 7), a full AT2 receptor agonist^65^, with the dose selected based on earlier reports^19,66^; Ang II (75 ng kg^−1^ min^−1^, *n* = 8), as previously discribed in mice, rats and sheep^67–69^; PD123319 (PD, 50 µg kg^−1^ min^−1^, *n* = 9), a selective AT2 receptor antagonist used at a dose established in prior work^70^; losartan (7.5 µg kg^−1^ min^−1^, *n* = 9), a selective AT1 receptor antagonist, as reported previously^13,68,71^ or Nitro-L-arginine methyl ester (L-NAME, 150 µg kg^−1^ min^−1^, *n* = 6), a nitric oxide synthase (NOS) inhibitor^13^.
2. *Ang II-infused groups*: Three groups received Ang II (75 ng kg^− 1^ min^− 1^) as the first I.C.V. infusion at a rate of 1 µl min^− 1^ for 2 min (loading dose), followed by a maintenance dose of 5 µl h^− 1^. The second I.C.V. infusion was then switched to either Ang II + L-NAME (75 ng kg^− 1^ min^− 1^ + 150 µg kg^− 1  ^min^−1^  *n* = 7), Ang II + PD123319 (75 ng kg^− 1^ min^− 1^ + 50 µg kg^− 1^min^−1^, *n* = 7) or Ang II + Losartan (75 ng kg^− 1^ min^− 1^ + 7.5 µg kg^−1^ min^−1^ , *n* = 9).
3. *CGP-infused group*: One group received CGP (50 µg kg^− 1 ^min^−1^) as the first I.C.V. infusion at a rate of 1 µl min^− 1^ for 2 min (loading dose), followed by a maintenance dose of 5 µl h^− 1^. The second I.C.V. infusion was then switched to CGP + L-NAME (50 µg kg^− 1^ + 150 µg kg^− 1^min^−1^ , *n* = 9).

**Fig. 5:**
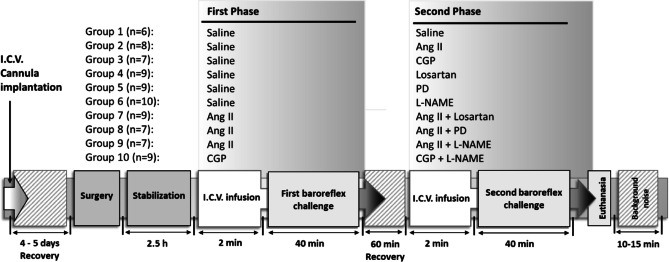
Schematic diagram summarizing experimental groups and the acute study protocol. Ang II, angiotensin II; CGP, CGP42112; I.C.V., intracerebroventricular; L-NAME, N^ω^-nitro-L-arginine methyl ester; MAP, mean arterial pressure; PD, PD123319.

The experimental groups and their respective treatments are detailed in Fig. 5.

## Data analysis

The analysis of the baroreflex curves for the relationship between MAP and integrated RSNA was performed by fitting data to a logistic sigmoid function [*y* = *A*1∕ (1 + exp (*A*2 (*x* − *A*3))) + *A*4], where *y* is RSNA , where *A*1 is the range between upper plateau (maximum RSNA response) and lower plateau, *A*2 defines the slope of the baroreflex curve, *A*3 is MAP at half the reflex range (BP50) and *A*4 is lower plateau which is the minimum RSNA ^34^. The averaged *A*1–*A*4 data for the group were used to plot a mean curve. Additional baroreflex gain curve variables were calculated according to the following equations as reported before^72^:

$$Maximal{\text{ }}gain\,=\, - \,A1 \times A2/4,$$

$$Maximum{\text{ }}response{\text{ }}={\text{ }}A1{\text{ }}+{\text{ }}A4,$$

$$MAP{\text{ }}threshold\,=\,-2.0/A2\,+\,A3,$$

$$MAP{\text{ }}saturation\,=\, \,2.0/A2\,+\,A3,$$

$$Operating{\text{ }}range\,=\,MAP{\text{ }}saturation\, - \,MAP{\text{ }}threshold.$$

Baroreflex sensitivity was assessed using two complementary measures: (1) slope (*A*2), which reflects the overall shape of the curve and provides a range-independent assessment of sensitivity and (2) maximum gain, which reflects the steepest part of the baroreflex curve and depends on the operating range^73^. To detect the differences between each of the baroreflex parameters in the first and second baroreflex curves, a two-tailed paired Student’s *t*-test was used for normally distributed data, whereas Wilcoxon matched-pairs signed rank test was applied for non-normally distributed data. Data were analysed offline and presented as mean ± SD, and values for *P* < 0.05 were accepted as statistically significant.

**Table 1:** Steady-state mean arterial pressure, heart rate, and renal sympathetic nerve activity obtained immediately before baroreflex gain curve generation in rats receiving various intracerebroventricular (I.C.V.) treatments. Values are expressed as means ± SD. *P < 0.05 s vs. first I.C.V. infusion. MAP, mean arterial pressure; HR, heart rate; RSNA, renal sympathetic nerve activity; Ang II, angiotensin II; LOS, Losartan; LNM, L-NAME; CGP, CGP42112; PD, PD123319.

	Steady-state parameters			
Group	MAP (mmHg)	HR (Beats min^− 1^)	RSNA (µV.s)	
*Saline/Saline* (*n* = 6)				
*Saline*	106 ± 19	394 ± 25	0.88 ± 0.49	
*Saline*	111 ± 11	419 + 14	0.85 ± 0.42	
*P value*	0.271	**0.019**	0.798	
*Saline/Ang II* (*n* = 8)				
*Saline*	106 ± 7	383 ± 28	1.50 ± 0.63	
*Ang II*	124 ± 11	399 ± 36	1.00 ± 0.33	
*P value*	**0.002**	0.238	**0.021**	
*Saline/CGP* (*n* = 7)				
*Saline*	110 ± 16	382 ± 38	1.76 ± 0.74	
*CGP*	110 ± 10	415 ± 21	1.98 ± 1.13	
*P value*	0.832	**0.031**	0.310	
*Saline/LOS* (*n* = 9)				
*Saline*	104 ± 9	371 ± 30	2.06 ± 0.47	
*LOS*	109 ± 12	383 ± 34	2.31 ± 1.03	
*P value*	0.262	0.304	0.375	
*Saline/PD* (*n* = 9)				
*Saline*	107 ± 9	388 ± 31	1.40 ± 0.81	
*PD*	110 ± 3	416 ± 23	1.76 ± 1.09	
*P value*	0.256	**0.022**	0.074	
*Saline/LNM* (*n* = 10)				
*Saline*	110 ± 11	379 ± 25	2.70 ± 2.50	
*LNM*	119 ± 10	420 ± 29	2.17 ± 1.60	
*P value*	**0.001**	**0.002**	0.375	
*Ang II/Ang II + LOS* (*n* = 9)				
*Ang II*	113 ± 16	375 ± 35	1.28 ± 0.91	
*Ang II + LOS*	110 ± 12	404 ± 25	1.90 ± 1.31	
*P value*	0.426	**0.004**	0.071	
*Ang II/Ang II + PD* (*n* = 7)				
*Ang II*	114 ± 9	351 ± 33	0.69 ± 0.24	
*Ang II + PD*	124 ± 13	357 ± 29	0.82 ± 0.56	
*P value*	**0.006**	0.586	0.773	
*Ang II/Ang II + LNM* (*n* = 7)				
*Ang II*	124 ± 6	359 ± 43	1.23 ± 0.98	
*Ang II + LNM*	132 ± 9	384 ± 50	1.05 ± 1.03	
*P value*	**0.004**	0.109	0.375	
*CGP/CGP + LNM* (*n* = 9)				
*CGP*	109 ± 13	394 ± 42	1.06 ± 0.48	
*CGP + LNM*	126 ± 17	414 ± 36	0.93 ± 0.49	
*P value*	**0.008**	**0.029**	0.627	

**Table 2:** Analysis of RSNA baroreflex curves obtained during background I.C.V. Infusion of saline, Ang IIand CGP . Max resp., maximum response; MAP thr, threshold pressure; MAP sat, saturation pressure; oper. range, operating range; max gain, maximum gain; Ang II, angiotensin II; LOS, Losartan; LNM, L-NAME; CGP, CGP42112.

		Baroreflex gain curve parameters							
Group	A1 (%)	A2 (mmHg^− 1^)	A3 (mmHg)	A4 (%)	Max resp. (%)	MAP ***thr*** (mmHg)	MAP ***sat*** (mmHg)	Oper. Range (mmHg)	Max gain (% mmHg^− 1^)
*Saline/Saline* (*n* = 6)									
*Saline*	112 ± 37	0.12 ± 0.03	118 ± 14	34 ± 11	146 ± 36	99 ± 15	136 ± 15	37 ± 10	−3.2 ± 1.2
*Saline*	123 ± 39	0.10 + 0.02	113 ± 9	34 ± 13	157 ± 32	92 ± 8	134 ± 12	42 ± 8	−3.0 ± 1.2
*P value*	0.522	0.098	0.268	0.985	0.398	0.156	0.701	**0.049**	0.604
*Saline/Ang II* (*n* = 8)									
*Saline*	108 ± 41	0.13 ± 0.09	112 ± 13	31 ± 23	138 ± 33	93 ± 15	130 ± 15	37 ± 14	−3.0 ± 0.9
*Ang II*	197 ± 34	0.10 ± 0.03	116 ± 11	29 ± 17	227 ± 29	95 ± 9	137 ± 14	42 ± 11	−5.0 ± 1.6
*P value*	**0.002**	0.883	0.338	0.641	**0.001**	0.787	0.087	0.430	**0.003**
*Saline/CGP* (*n* = 7)									
*Saline*	109 ± 22	0.12 ± 0.04	111 ± 17	34 ± 21	143 ± 22	94 ± 16	128 ± 19	34 ± 8	−3.3 ± 0.6
*CGP*	154 ± 27	0.09 ± 0.02	107 ± 12	22 ± 16	176 ± 34	84 ± 16	131 ± 11	47 ± 11	−3.4 ± 0.7
*P value*	**0.029**	0.109	0.469	**0.017**	0.056	0.156	0.375	0.078	0.337

**Table 3:** Analysis of RSNA baroreflex curves obtained during background I.C.V. Infusion of saline, LOS, PD and LNM. . Max resp., maximum response; MAP thr, threshold pressure; MAP sat, saturation pressure; oper. Range, operating range; max gain, maximum gain; LOS, Losartan; LNM, L-NAME.

		Baroreflex gain curve parameters							
Group	A1 (%)	A2 (mmHg^− 1^)	A3 (mmHg)	A4 (%)	Max resp. (%)	MAP ***thr*** (mmHg)	MAP ***sat*** (mmHg)	Oper. Range (mmHg)	Max gain (% mmHg^− 1^)
*Saline/LOS* (*n* = 9)									
*Saline*	131 ± 24	0.09 ± 0.03	109 ± 10	21 ± 14	152 ± 25	85 ± 17	133 ± 7	47 ± 17	−3.0 ± 0.9
*LOS*	157 ± 53	0.13 + 0.05	107 ± 5	27 ± 11	184 ± 58	89 ± 5	125 ± 9	35 ± 11	−4.8 ± 1.9
*P value*	0.082	**0.037**	0.570	0.162	0.056	0.395	**0.012**	**0.020**	**0.004**
*Saline/PD* (*n* = 9)									
*Saline*	121 ± 42	0.13 ± 0.09	111 ± 10	23 ± 7	144 ± 39	91 ± 15	131 ± 10	40 ± 16	−3.4 ± 1.3
*PD*	119 ± 45	0.07 ± 0.01	113 ± 10	34 ± 15	153 ± 33	83 ± 12	142 ± 10	59 ± 9	−2.0 ± 0.8
*P value*	0.856	**0.004**	0.337	**0.025**	0.249	**0.006**	**0.007**	**0.002**	**0.003**
*Saline/LNM* (*n* = 10)									
*Saline*	121 ± 43	0.11 ± 0.06	112 ± 12	31 ± 27	152 ± 37	90 ± 15	135 ± 17	45 ± 22	−3.1 ± 1.5
*LNM*	131 ± 47	0.11 ± 0.03	120 ± 12	32 ± 21	162 ± 36	99 ± 15	140 ± 13	41 ± 14	−3.4 ± 1.4
*P value*	0.606	0.737	**0.025**	0.922	0.506	0.091	0.196	0.592	0.339

**Table 4:** Analysis of RSNA baroreflex curves obtained during background I.C.V. Infusion of saline, Ang IIand CGP . Max resp., maximum response; MAP thr, threshold pressure; MAP sat, saturation pressure; oper. range, operating range; max gain, maximum gain; Ang II, angiotensin II; LOS, Losartan; LNM, L-NAME; CGP, CGP42112.

		Baroreflex gain curve parameters							
Group	A1 (%)	A2 (mmHg^− 1^)	A3 (mmHg)	A4 (%)	Max resp. (%)	MAP ***thr*** (mmHg)	MAP ***sat*** (mmHg)	Oper. Range (mmHg)	Max gain (% mmHg^− 1^)
*Ang II/Ang II + LOS* (*n* = 9)									
*Ang II*	168 ± 49	0.11 ± 0.04	113 ± 14	18 ± 13	186 ± 47	92 ± 13	138 ± 19	41 ± 18	−4.7 ± 2.5
*Ang II + LOS*	160 ± 55	0.12 + 0.06	107 ± 10	18 ± 8	178 ± 52	87 ± 14	127 ± 13	40 ± 18	−4.6 ± 1.9
*P value*	0.725	0.482	0.145	0.991	0.910	0.336	0.245	0.808	0.460
*Ang II/Ang II + PD* (*n* = 7)									
*Ang II*	180 ± 48	0.12 ± 0.03	111 ± 12	29 ± 23	209 ± 39	92 ± 15	129 ± 10	37 ± 9	−4.9 ± 0.4
*Ang II + PD*	163 ± 37	0.09 ± 0.03	118 ± 8	33 ± 19	196 ± 33	95 ± 10	140 ± 11	46 ± 13	−3.7 ± 1.1
*P value*	0.189	**0.034**	**0.009**	0.688	0.438	0.340	**0.016**	**0.050**	**0.031**
*Ang II/Ang II + LNM* (*n* = 7)									
*Ang II*	188 ± 60	0.11 ± 0.06	117 ± 6	35 ± 21	223 ± 47	96 ± 11	138 ± 9	42 ± 15	−4.7 ± 0.9
*Ang II + LNM*	180 ± 32	0.10 ± 0.04	124 ± 11	35 ± 34	215 ± 31	101 ± 15	146 ± 14	45 ± 19	−4.6 ± 1.6
*P value*	0.615	0.469	0.164	> 0.999	0.685	0.578	0.143	0.738	0.839
*CGP/CGP + LNM* (*n* = 9)									
*CGP*	141 ± 32	0.09 ± 0.03	115 ± 15	19 ± 7	160 ± 28	90 ± 18	140 ± 16	50 ± 16	−3.0 ± 1.0
*CGP + LNM*	133 ± 35	0.11 ± 0.03	120 ± 18	35 ± 15	168 ± 32	101 ± 18	140 ± 18	39 ± 10	−3.5 ± 1.2
*P value*	0.570	**0.045**	0.354	**0.013**	0.820	0.164	0.946	**0.035**	**0.020**

## Data Availability

The data that support the findings of this study are available on request from the corresponding author.
